# Effect of sage (*Salvia officinalis* L.) extract in antioxidant status and intestinal morphology of pulmonary hypertensive chickens

**DOI:** 10.1002/vms3.804

**Published:** 2022-04-11

**Authors:** Shahab Bahadoran, Younes Teymouri, Hossein Hassanpour, Abdolnaser Mohebbi, Mohammad Reza Akbari

**Affiliations:** ^1^ Department of Clinical Sciences Faculty of Veterinary Medicine Shahrekord University Shahrekord Iran; ^2^ Department of Basic Sciences Physiology Division Faculty of Veterinary Medicine Shahrekord Iran; ^3^ Department of Animal Science Faculty of Agriculture Shahrekord University Shahrekord Iran

**Keywords:** ascites, broiler, digestive system, herbal plant

## Abstract

**Objectives:**

The effects of dietary sage on the growth performance, antioxidant status, intestinal mucosa morphology, and pulmonary hypertensive response were investigated in broiler chickens with pulmonary hypertension.

**Methods:**

Chicks (Ross 308) were reared under cold stress for 35 days and treated with 0.05% vitamin C (positive control) and 0 (control), 0.1 or 0.2% sage extracts, then performance, oxidant and antioxidant status, and intestinal morphology were evaluated.

**Results:**

The index of pulmonary hypertension (RV:TV) was decreased, and weight gain (days 22–35) was increased in all treatments (except for sage 0.1%) compared with control (*P < *0.05). Lipid peroxidation was decreased, whereas the activity of antioxidant enzymes (GPX, CAT, and SOD) was increased in the sage 0.2% group compared with control (*P* < 0.05). In the lung, SOD, CAT, and GPX transcripts were decreased in the sage 0.2% group compared with control (*P* < 0.05). In the right ventricle of the heart, SOD and CAT transcripts were increased in the sage 0.2% group compared with other groups of chickens, whereas GPX transcript was decreased (*P* < 0.05). The jejunal villus length in the chickens fed sage was significantly lower than in control (*P < *0.05). The ileal villus width, villus surface area, and lamina proporia thickness in the chickens fed sage (0.2%) were increased compared with control (*P *< 0.05).

**Conclusions:**

Dietary supplementation of sage (0.2%) could modulate pulmonary hypertensive response, improve antioxidant status (enzymatic activity), intestinal morphometry, and absorptive surface in the broiler chickens.

## Graphical abstract

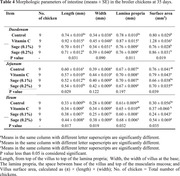

## 1 INTRODUCTION

Herbal plants are among the principal sources of human and animal drugs and play an important role in the world health care systems. A growing interest has been recently developed in the area of animal nutrition and in the potential impact of medicinal plants and herbs on the growth performance and treatment of diseases (Levkut et al., 2010). Herbal plant extracts had been applied in times before Christ in the Middle East to treat different disorders. Among these herbs, sage (*Salvia officinalis* L.), a plant belonging to the Lamiaceae family, is a native aromatic and medicinal plant and grows in Iran and countries bordering the Mediterranean Sea (Miliauskas et al., 2004). Sage extract consists of rosmarinic and carnosic acids at high concentrations, which have antioxidant properties (Yurtseven et al., 2008). It has been confirmed that leaves, roots, and/or water‐soluble extractions of S. *officinalis* L. contain 1%–2.5% volatile fatty acids, saponins, diterpenes, flavonoids, phenolic acids, salviatannins, resin and estrogenic substances (Taarit et al., 2010). Sage essential oils have been suggested in the treatment of several diseases in the nervous, cardiovascular, respiratory, digestive, metabolic, and endocrine systems (Hamidpour, 2015). Pharmacological knowledge about sage has risen in recent decades, and its useful effects as antibacterial, antioxidant, anti‑inflammatory, free radical scavenging, and antitumor activities have been reported. It is recommended that sage could be used in the production of novel natural drugs to prevent, control, and treat many dangerous and complicated diseases such as diabetes, Alzheimer's, and cancer. In addition, sage essential oil has been shown to have carminative, antispasmodic, antiseptic, and astringent properties (Hamidpour et al., 2014).

In many experiments, sage has been supplemented with bird diets to understand the impacts on the growth and feed conversion (Levkut et al., 2010; Yurtseven et al., 2008). Many of these experiments showed positive effects of these plants on the mentioned parameters, while other reports concluded that sage had no influence on the growth and feed conversion.

Because of the beneficial effects of sage in different diseases and their antioxidant effect, it would be permissible that this medicinal plant affects one of the serious metabolic disorders in broiler chickens—pulmonary hypertension syndrome (PHS; ascites). In this syndrome of chicken, the fast‐growth property plays a key role in the rise of tissue demand of oxygen and cardiac output. The vascular capacity of lungs in the chickens is anatomically unable to respond to this raised cardiac output. Then, the resistance of pulmonary vasculature increases, which leads to an imbalance between pulmonary vasoconstrictors and vasodilators (Hassanzadeh et al., 2013; Wideman et al., 2013). This process finally results in hypoxemia, right‐sided congestive heart failure, central venous congestion, cirrhosis of the liver, and accumulation of ascitic fluid into the abdominal cavity (Biswas, 2019). One of the critical pathophysiologic factors in PHS is free radicals. Broilers developing PHS showed the high amounts of free radicals in the cardiopulmonary tissues, which led the disease to be worsened (Biswas, 2019; Singh et al., 2018). Previous studies demonstrated the negative effect of free radicals on the cardiovascular and gastrointestinal systems of pulmonary hypertensive chickens (Baghbanzadeh & Decuypere, 2008; Janwari et al., 2018). We hypothesize that sage with its useful effects may modulate the pulmonary hypertensive response due to cold stress. We investigate whether sage extracts via its antioxidant effects could reduce ascites incidence and improve growth performance through change of intestinal morphology.

## 2 MATERIALS AND METHODS

### 2.1 Plant preparation and extraction

Sage plant used in this experiment was collected in the summer when the plant was in the vegetative stage, in Chahar Mahalo Bakhtiari province, Iran. Collected leaves were shadow dried and ground with a laboratory hammer mill. To prepare sage extracts, dried leaf powders were mixed with methanol 80% with a 2:10 ratio. Then, they were shaken for 15 min to be completely mixed, kept for 24 h at room temperature, passed through a Whatmann filter paper No. 1, and then dried in 40°C for 48 h. The amount of 11 g extract was prepared from each 100 g sage powder. Identification of chemical constituents of the sage extract was performed by gas chromatography–mass spectrometry.

### 2.2 Animals, management, and treatments

One hundred forty‐four one‐day‐old broiler chickens (Ross 308) were randomized across 18 floor pens measuring 1.5 m × 1 m × 1 m each. Chicks were assigned to one of four groups (two controls and two treatments) with three replicates pens per group and 12 chickens per pen. Chickens were housed in a deep litter system with wood shaving. Chicks were reared in standard conditions (temperature, ventilation, and light) for 35 days and had free access to water and feed. A standard basal diet in mash form was formulated for the starting (1–10 days), growing (11–24 days), and finishing (25–35 days) growth stages, which were mainly composed of corn and soybean meal (Aviagen, 2018). For the treatments, sage was included in the starter, grower, and finisher basal diets at concentrations of 0.1, and 0.2% of the diet. To induce PHS, cold stress was used for all groups of chickens in the rearing room, according to a temperature program offered by Teshfam et al. (2006a) and Hassanpour et al. (2015b) with little modification (Table 1). In each pen, the amount of feed offered and refused was weighed and recorded every day to calculate intake. Feed consumption (FC) and body weight were recorded per pen, and then weight gain (WG) and feed conversion rate (FCR) were computed. During the rearing period, mortality was recorded daily, and dead broilers were examined for lesions of ascites.

**TABLE 1:** Cold induction for all groups of chickens (compared with standard temperature)

	Age (day) of chickens											
	1	3	5	7	9	11	13	15	17	19	21	23–35
Standard condition	32	31	30	29	28	27	26	25	24	23	22	21–22
Cold‐induced PHS	32	31	30	29	25	22	19	18	17	16	16	14–15

PHS = pulmonary hypertension syndrome.

### 2.3 Assessment of pulmonary hypertension index

At the end of the feeding trial (35 days of age), all chickens from each group were euthanized by decapitation (Harash et al., 2019; Wang et al., 2019). The heart and then ventricles were dissected and weighed to calculate the right ventricle to total ventricle ratio (RV:TV ratio). Right ventricle hypertrophy and pulmonary hypertension were estimated by this index as described by previous studies (Ahmadipour et al., 2019; Hassanpour et al., 2011). According to this index, chickens with an RV/TV ratio of more than 0.25 were reported to have developmental pulmonary hypertension, whereas chickens with an RV:TV ratio ≥ 0.29 were found to show clinical PHS, i.e., ascites with considerable right ventricular dilation (Author et al., 2016). Morbidity of PHS is determined after killing of all chickens at 35 days according to the ratio > 0.25.

### 2.4 Morphometric analysis of the intestine

At 35 days of age, 9 chicks were selected randomly from each group (3 chickens from each pen), decapitated, and their morphometric variables of intestine (villus length, width, surface area, and lamina propria thickness) were evaluated according to the method of Teshfam et al. (2006b) and Hassanpour et al. (2016). Briefly, midpoint segments of the duodenum, jejunum, and ileum were fixed in Clark fixative solution and ethyl alcohol. Each segment was stained with periodic acid–Schiff stain, and then villus rows were cut in sagittal part of the sections and placed between a glass slide and cover‐slip. The villus dimensions were measured by using a light microscope with an eyepiece graticule. The villus length was estimated from the top of the villus to the top of the lamina propria. The villus surface area was calculated as (π) × (villus width) × (villus length). The lamina propria thickness as a place of lieberkuhn glands was measured from the base of the villus and the top of the muscularis mucosa.

### 2.5 Measurement of MDA and CAT, GPX, and SOD enzyme activities

The catalase (CAT), glutathione peroxidase (GPX), superoxide dismutase (SOD) activities (indicators of enzymatic antioxidant defense system), and malondialdehyde (MDA, an indicator of lipid peroxidation) were assayed according to Hassanpour et al. (2015a) and (Ahmadipour et al., 2021) in the serum samples of nine chickens from each group at 35 days. One unit of CAT activity was defined as the amount of enzyme that catalyzes the decomposition of 1 mol of hydrogen peroxide per minute per total serum protein. One unit of GPX activity was reported as μmol NADPH consumed per minute per milligram total serum protein of sample, using the appropriate molar absorptivity coefficient for NADPH. The results for SOD activity were expressed as the percentage of inhibition of reduction in NBT by SOD per total serum protein of sample.

### 2.6 RNA extraction, cDNA synthesis, and quantitative real‐time PCR analysis of lung and heart tissues

Lung and heart (right ventricle) tissues were processed for total RNA isolation by RNX‐Plus solution (Sinaclon Bioscience, Karaj, Iran) according to Pirany et al. (2020). The RNase‐free DNase (Sinaclon Bioscience, Iran) was added to the extracted RNA to remove contaminating genomic DNA. Only RNA samples exhibiting an A260/A280 ratio of more than 1.9 were used for the cDNA synthesis. PrimeScript TM RT reagent kit (Takara Bio Inc., Japan) was used to synthesize cDNA according to a previous study (Hassanpour et al., 2019).

The specific primers of CAT, GPX, SOD, and YWHAZ (as an endogenous standard) (Hassanpour et al., 2018) were prepared according to Hassanpour et al. (2015a) to determine their relative amounts by quantitative real‐time polymerase chain reaction (PCR). SYBR PremixExTaq II (Tli RNaseH Plus) kit (Takara BioInc., Japan) was used to do PCR, and the thermal profile was 40 cycles of 95°C for 40 s, 66°C for 30 s, and 72°C for 30 s (Ahmadipour et al., 2015; Hassanpour et al., 2015b). The mRNA level of each target gene relative to YWHAZ was estimated for each sample with Pfaffl method as described previously by Hassanpour et al. (2015b).

### 2.7 Statistical analysis

Data are represented as mean of each group. Comparisons were made using one‐way ANOVA with Duncan's multiple range test between different groups of the experiment. All statistical analyses were performed with the Statistical Package for Social Sciences software version 17 (SPSS Inc, Chicago, IL, USA). *P* values less than 0.05 were considered significant.

## 3 RESULTS

### 3.1 Sage compositions

Table 2 represents the major compositions of sage. Specific constituents of β‐thujone, 1,8‐cineole, camphor, α‐thujone, borneol, α‐humulene, and α‐pinene were mostly present in the leaf extract.

**TABLE 2:** The major compositions of leaf extract of *Salvia officinalis* L

Constituents	Percentage	Constituents	Percentage
β‐Thujone	10.8	Humulene epoxide II	0.9
1,8‐Cineole	8.3	ϒ‐Terpinene	0.7
Camphor	6.3	Bornyl actate	0.5
α‐Thujone	4.5	β‐Pinene	0.3
Borneol	3.9	Caryophyllene oxide	0.2
α‐Humulene	3.7	Limonene	0.2
α‐Pinene	2.4	Myrcene	0.2
Camphene	2.2	p‐Cymene	0.1
Glubulol	2.2	Terpinolene	0.1
Methyl octadecanoate	1.8	Terpinen‐4‐ol	0.1
ϒ‐Murolene	1.7	Trans‐pinocarveol	0.1
Manool	1.7	Diterpenes	0.1
Linalool	1.4	Sesquiterpenes	1.3
Trans‐caryophyllene	1.2	Monoterpenes	8.7
Aromadendrene	1.0	Oxygenated monoterpenes	26.4

### 3.2 Index of PHS and mortality rate

The RV:TV ratio as an index of PHS ratio was decreased to less than 0.25 in vitamin C (0.23 ± 0.017) and sage (0.2%) (0.21 ± 0.026) groups of chickens compared with control group (0.35 ± 0.035) at 35 days of age (*P* < 0.05). The RV:TV ratio was not significant between different treated groups (*p* > 0.05). The RV:TV ratio with an amount of 0.29 ± 0.031 in the sage (0.1%) group of chickens was also not significant among all groups (*p* > 0.05). The morbidity rates of PHS were 72%, 35%, 55%, and 40% in the control, vitamin C, sage 0.1%, and sage 0.2% groups of chickens, respectively. Ascites mortality was decreased in vitamin C and sage 0.2% groups of chickens compared with the control and sage 0.1% groups (Table 3).

**TABLE 3:** Broiler growth performance (means ± SE) of the experimental groups

Item	No. of chickens	WG (g)	FC (g)	FCR	Mortality (%)
*Days 3 to 21*					
Control	36	671.8 ± 8.91^a^	1,048.4 ± 11.71^a^	1.56 ± 0.47^a^	–
Vitamin C	36	666.4 ± 8.11^a^	986.2 ± 10.54^a^	1.48 ± 0.51^a^	–
Sage (0.1%)	36	677.2 ± 8.57^a^	1,002.3 ± 10.79^a^	1.48 ± 0.41^a^	–
Sage (0.2%)	36	642.7 ± 7.98^a^	976.9 ± 9.78^a^	1.52 ± 0.57^a^	–
*P* value	–	0.231	0.410	0.103	–
*Days 22 to 35*					
Control	36	855.8 ± 8,88^b^	1,890.8 ± 12.10^a^	2.21 ± 0.17^a^	–
Vitamin C	36	1,018.8 ± 9.45^a^	1,976.6 ± 11.87^a^	1.94 ± 0.09^a^, ^b^	–
Sage (0.1%)	36	958.2 ± 9.11^a^, ^b^	1,935.5 ± 10.65^a^	2.02 ± 0.22^a^, ^b^	–
Sage (0.2%)	36	1,038.1 ± 9.97^a^	2,045.2 ±15.87 ^a^	1.97 ± 0.15^a^, ^b^	–
*P* value	–	0.043	0.097	0.037	–
*Days 3 to 35*					
Control	36	1,544.2 ± 8.90^a^	2,980.2 ± 11.98^a^	1.93 ± 0.34^a^	12.5^a^
Vitamin C	36	1,591.4 ± 9.54^a^	2,896.3 ± 11.67^a^	1.82 ± 0.32^a^	5.6^b^
Sage (0.1%)	36	1,672.8 ± 9.01^a^	2,945.8 ± 10.70^a^	1.76 ± 0.29^a^	9.0^a^ ^c^
Sage (0.2%)	36	1,646.1 ± 9.43^a^	2,864.3 ± 13.98^a^	1.74 ± 0.38^a^	7.6^b^ ^c^
*P* value	–	0.289	0.187	0.302	0.041

^a^ Means in the same column with different letter superscripts are significantly different.

^b^ Means in the same column with different letter superscripts are significantly different.

*P* value less than 0.05 is considered significant.

Abbreviations: FC = feed consumption; FCR = feed conversion ratio; No. of chickens = Total number of chickens, and 12 chickens/pen at each time; WG = weight gain.

### 3.3 Growth performance

The growth performance of chickens in the all experimental groups is given in Table 2. The initial body weight did not differ between experimental groups (data not shown). Growth performance (WG, FC, and FCR) of the chickens was not significantly different between experimental groups at 3–21 and 3–35 days of rearing period. The WG of chickens in all treatments (except for sage 0.1%) was increased in days 22–35 compared with control (*P* < 0.05). FC did not differ among groups, and FCR and WG were not significant between vitamin C and sage groups of chickens (*p* > 0.05; Table 3).

### 3.4 Morphometric assessment of intestine

Morphologic parameters of intestinal villi in the different groups of chickens were dedicated in Table 4. The duodenal villus length, lamina propria thickness, and surface area in the chickens fed vitamin C were higher than in control (*P* < 0.05). The jejunal villus length in the chickens fed sage was significantly lower than in control. The jejunal and duodenal villus width was similar between experimental groups.The ileal villus width in the chickens fed vitamin C and sage (0.2%), and villus surface area / lamina proporia thickness in the chickens fed sage (0.2%) were increased compared with control (*P* < 0.05).

**TABLE 4:** Morphologic parameters of intestine (means ± SE) in the broiler chickens at 35 days

Item	No. of chicken	Length (mm)	Width (mm)	Lamina propria (mm)	Surface area (mm^2^)
*Duodenum*					
Control	9	0.74 ± 0.010^b^, ^c^	0.34 ± 0.038^a^	0.78 ± 0.010^b^, ^c^	0.80 ± 0.029^b^
Vitamin C	9	0.92 ± 0.015^a^	0.45 ± 0.048^a^	0.87 ± 0.015^a^	1.28 ± 0.036^a^
Sage (0.1%)	9	0.70 ± 0.011^b^	0.34 ± 0.031^a^	0.76 ± 0.009^c^	0.76 ± 0.028^b^
Sage (0.2%)	9	0.71 ± 0.012^b^	0.39 ± 0.040^a^	0.76 ± 0.009^c^	0.86 ± 0.031^b^
*P* value	–	0.031	0.090	0.011	0.019
*Jejunum*					
Control	9	0.60 ± 0.016^a^	0.39 ± 0.008^a^	0.67 ± 0.007^a^	0.76 ± 0.041^a^, ^b^
Vitamin C	9	0.57 ± 0.010^a^, ^c^	0.44 ± 0.010^a^	0.70 ± 0.008^a^	0.79 ± 0.043^a^
Sage (0.1%)	9	0.52 ± 0.012^b^, ^c^	0.40 ± 0.009^a^	0.70 ± 0.007^a^	0.66 ± 0.038^b^, ^c^
Sage (0.2%)	9	0.54 ± 0.010^b^, ^c^	0.42 ± 0.009^a^	0.67 ± 0.009^a^	0.70 ± 0.035^a^, ^c^
*P* value		0.029	0.122	0.197	0.039
*Ileum*					
Control	9	0.33 ± 0.009^b^, ^c^	0.28 ± 0.008^c^	0.61± 0.009^b^	0.30 ± 0.056^b^, ^c^
Vitamin C	9	0.34 ± 0.009^b^	0.34 ± 0.009^a^	0.65 ± 0.010^a^, ^b^	0.37 ±0.060 ^b^
Sage (0.1%)	9	0.38 ± 0.007^c^	0.25 ± 0.007^c^	0.60 ± 0.008^c^	0.24 ± 0.043^c^
Sage (0.2%)	9	0.44 ± 0.008^a^	0.38 ± 0.009^a^	0.68 ± 0.006^a^	0.54 ± 0.069^a^
*P* value	–	0.017	0.019	0.032	0.035

^a^ Means in the same column with different letter superscripts are significantly different.

^b^ Means in the same column with different letter superscripts are significantly different.

^c^ Means in the same column with different letter superscripts are significantly different.

*P* value less than 0.05 is considered significant.

Length, from top of the villus to top of the lamina propria; Width, the width of villus at the base; The lamina propria, the space between base of the villus and top of the muscularis mucosa; and Villus surface area, calculated as (π) × (length) × (width); No. of chicken = Total number of chickens.

### 3.5 Assessment of lipid peroxidation and SOD, CAT, and GPX activities

Results of lipid peroxidation (MDA measurement) and antioxidant enzyme activities in the serum samples of chickens are presented in Table 5. MDA was decreased, and GPX was increased in all the treatments compared with control (*P* < 0.05), whereas CAT and SOD were increased only in sage 0.2% and vitamin C groups compared with control, respectively (*P* < 0.05).

**TABLE 5:** Plasma biochemical analysis (means ± SE ) of oxidant and antioxidant status in broiler chickens at 35 days

	No. of chicken	MDA (μmol/mL)	CAT (U/mg protein)	SOD (% inhibition)	GPX (U/mg protein)
**Control**	9	36.1 ± 5.40^b^	107.4 ± 8.92^a^	17.1 ± 3.22^a^, ^c^	12.1 ± 2.63^a^
**Vitamin C**	9	22.3 ± 4.11^a^	111.1 ± 9.31^a^	28.1 ± 4.41^b^	18.3 ± 3.72^b^
**Sage (0.1%)**	9	18.1 ± 3.83^a^	130.2 ± 10.31^a^, ^b^	19.9 ± 3.80^a^, ^b^	17.4 ± 2.93^b^
**Sage (0.2%)**	9	23.7 ± 5.11^a^	162.2 ± 11.8^b^	26.3 ± 4.21^b^, ^c^	18.5 ± 3.94^b^
**P value**	–	0.010	0.018	0.031	0.009

^a^ Means in the same column with different letter superscripts are significantly different.

^b^ Means in the same column with different letter superscripts are significantly different.

^c^ Means in the same column with different letter superscripts are significantly different.

*P* value less than 0.05 is considered significant.

Abbreviations: CAT = catalase; GPX = glutathione peroxidase; MDA = malondialdehyde; SOD = superoxide dismutase.

### 3.6 Relative expression of SOD, CAT, and GPX genes

The amounts of relative expression of SOD, CAT, and GPX genes in the lung and heart tissues are presented in Table 6. In the lung, relative amounts of SOD and GPX genes were decreased in the sage 0.2% group compared with other groups of chickens (*P* < 0.05). The amount of CAT gene was also decreased in the sage 0.2% group compared with vitamin C and control groups (*P* < 0.05). In the right ventricle of heart, relative amounts of SOD and CAT genes were increased in the sage 0.2% group compared with other groups of chickens (*P* < 0.05), whereas the amount of the GPX gene was decreased in the sage (0.2%) compared with other groups (*P* < 0.05). The relative expression of these three genes did not change in the sage 0.1% group of chickens compared with control and Vit C (*P *> 0.05).

**TABLE 6:** Relative expression (target/YWHAZ; means ± SE) of chicken genes at 35 days

	No. of chicken	CAT	SOD	GPX
Lung				
Control	9	0.09 ± 0.006^a^	3.75 ± 0.221^a^	4.65 ± 0.982^a^
Vitamin C	9	0.10 ± 0.008^a^	4.16 ± 0.391^a^	5.63 ± 0.865^a^
Sage (0.1%)	9	0.08 ± 0.008^a^, ^b^	3.30 ± 0.251^a^	4.24 ± 0.767^a^
Sage (0.2%)	9	0.05 ± 0.005^b^	1.18 ± 0.165^b^	1.06 ± 0.391^b^
*P* value	–	0.008	0.012	0.018
Heart (right ventricle)				
Control	9	0.19 ± 0.209^a^	1.31 ± 0.787^a^	4.39 ± 0.908^a^
Vitamin C	9	0.27 ± 0.287^a^	3.67 ± 0.911^a^	3.35 ± 0.686^a^
Sage (0.1%)	9	0.27 ± 0.211^a^	2.33 ± 0.730^a^	4.96 ± 0.851^a^
Sage (0.2%)	9	1.83 ± 0.114^b^	6.24 ± 0.856^b^	1.76 ± 0.337^b^
*P* value	–	0.021	0.006	0.009

^a^ Means in the same column with different letter superscripts are significantly different.

^b^ Means in the same column with different letter superscripts are significantly different.

*P* value less than 0.05 is considered significant.

Abbreviations: CAT = catalase; GPX = glutathione peroxidase; MDA = malondialdehyde; SOD = superoxide dismutase.

## 4 DISCUSSION

In the present study, vitamin C was used as a positive control. Its beneficial effects have already been confirmed in the chicken growth performance, antioxidant activity, pulmonary hypertensive response, and intestinal morphology in previous studies (El‐Senousey et al., 2018; Zamani Moghaddam et al., 2009). Then, the comparison of vitamin C effects with sage could be useful.

Results based on the RV:TV ratio showed that sage could modulate pulmonary hypertensive response and decrease developmental hypertrophy and dilation of the heart in broilers. Chen and Chen (2017) explained that the phenolic acids and tanshinones target multiple signaling pathways in a variety of tissues to ameliorate atherosclerosis, thrombosis, and myocardial reperfusion injury. Alshubaily and Jambi (2018) also reported that essential oil obtained from sage has medicinal effects against respiratory, heart, blood circulation, and metabolic conditions. Although early report has shown that sage improves body weight gain, food consumption, or feed conversion ratio, in the chickens, there is also conflicting report (Yurtseven et al., 2008). In studies on this plant, different products (oil, essential oil, powder, and extract) with different concentrations have been used. The main compounds and characteristics of sage are different in these products and may considerably change their functions in the mentioned studies. In our study, two concentrations of sage did not influence the performance at all sampling times of the rearing period, which is consistent with many studies reporting no effect of sage on body performance (Levkut et al., 2010; Yurtseven et al., 2008).

As noted, one of the factors involved in the pathogenesis of PHS is oxidants. To protect the cells and organs against oxidants, there is a complex antioxidant protection system that neutralizes and scavenges free radicals. Enzymatic antioxidants (such as SOD, CAT, and GPX) are the important members of this protection system that play a crucial role in the cellular defense. SOD, CAT, and GPX constitute a mutually supportive team of defense against oxidant. While SOD lowers the steady‐state level of O2^–^, CAT and GPX do the same for H_2_O_2_ (Surai et al., 2019). It has been confirmed that exposure to low temperatures could damage the cell antioxidant and protective (heat shock proteins) systems (Zhao et al., 2013). Our evaluation showed an increase of total antioxidant and decrease of oxidant status in the hypertensive chickens fed sage, especially in concentration of 0.2%. Different studies have been determined that compounds such as thujone, 1,8‐cineole, camphor, borneol, α‐humulene, and oxygenated monoterpenes have strong antioxidative, anti‐inflammatory, and antibacterial effects (Badawy et al., 2019; Bansod et al., 2021; Ciftci et al., 2011; Shata et al., 2014; Srinivasan et al., 2020; Yeo et al., 2021). These compounds were considered major constituents of sage leaf extract in our study. Thus, this strong effect of sage constituents in the oxidant and antioxidant status could justify its improvement in the developmental PHS and its other following advantages in the intestine.

The lung and heart are two main organs in PHS. Our study reported that the effect of sage in the gene expression of antioxidant enzymes in these organs is varied. While these genes were downregulated in the lung, they were downregulated and upregulated in the heart. This condition has also been reported in other studies (Hassanpour et al., 2015a; Vongsak et al., 2015), leading to the fact that gene regulation of cells are complicated, and various factors involve in the control of genes. Also, different negative and positive cellular feedbacks could influence the gene regulatory pathway. On the other hand, the rate of oxidant production is varied in the different cells, then transcriptional activity and also mRNA stability would be different in each cell (Allen & Tresini, 2000). This may justify different acts of sage in the gene expression of the lung and heart.

Solis de los Santos et al. (2005) and Zamani Moghaddam et al. (2009) reported that PHS was associated with a progressive impairment of gut architecture and function. Many studies have examined different additives and supplements (e.g., vitamin C, L‐arginine, garlic) in the diets of pulmonary hypertensive chickens to modulate PHS and improve gut function (Author et al., 2016; Sharifi et al., 2015; Zamani Moghaddam et al., 2009).

In our study, we determined that oral supplementation of sage extracts affected intestinal morphology and improved villus dimensions in pulmonary hypertensive broilers. The effect of sage on the villus architecture was noticeable in the ileum. This effect of sage only in the ileum may be due to different mechanisms of its absorption in the intestine, leading it to ileum. The improvement of sage on the villus dimensions, especially the villus surface area, could represent a better condition in the ileum for nutrient absorption. The beneficial effects of sage on the intestine may be associated with its antioxidant and antiapoptotic effects (El‐Wakf et al., 2020; Roby et al., 2013) as it has previously been shown that dietary antioxidants such as vitamin C protect epithelial cells of intestine against proapoptotic oxidant stress, which increase epithelial cell growth and villus dimensions (Miller et al., 2001; Zamani Moghaddam et al., 2009). Therefore, the antioxidant effect of sage could be an important factor to protect the intestine against oxidative damage in PHS. Certainly, this effect is not limited to the intestine; it may also refer to heart, lung, and circulation that are crucial organs in PHS.

## 5 CONCLUSION

Dietary supplementation of sage could modulate pulmonary hypertensive response and improve (especially concentration of 0.2% sage) the intestinal morphometry and absorptive surface in the broiler chickens by activating the enzymatic antioxidant system.

## AUTHOR CONTRIBUTIONS

Conceptualization: SS, MRA. Data curation: YT, SS. Formal analysis: HH, YT, SS. Investigation: SS, HH. Methodology: AM, HH. Supervision: SS. Writing manuscript: HH, SS.

## CONFLICT OF INTEREST

The authors declare no potential conflict of interest.

## ETHICS STATEMENT

This experiment was approved by the Institutional Animal Care and Use Committee of Shahrekord University (letter No. IR.SKU.REC.1399.0876) in accordance with the standard of 1964 Declaration of Helsinki.

### PEER REVIEW

The peer review history for this article is available at https://publons.com/publon/10.1002/vms3.804

## Data Availability

Data are available upon request.
